# FRUIT, a Scar-Free System for Targeted Chromosomal Mutagenesis, Epitope Tagging, and Promoter Replacement in *Escherichia coli* and *Salmonella enterica*

**DOI:** 10.1371/journal.pone.0044841

**Published:** 2012-09-27

**Authors:** Anne M. Stringer, Navjot Singh, Anastasiya Yermakova, Brianna L. Petrone, Jayaleka J. Amarasinghe, Lucia Reyes-Diaz, Nicholas J. Mantis, Joseph T. Wade

**Affiliations:** 1 Wadsworth Center, New York State Department of Health, Albany, New York, United States of America; 2 Department of Biomedical Sciences, University at Albany, Albany, New York, United States of America; 3 UASRP CSTEP Program, University at Albany, Albany, New York, United States of America; University of Birmingham, United Kingdom

## Abstract

Recombineering is a widely-used approach to delete genes, introduce insertions and point mutations, and introduce epitope tags into bacterial chromosomes. Many recombineering methods have been described, for a wide range of bacterial species. These methods are often limited by (i) low efficiency, and/or (ii) introduction of “scar” DNA into the chromosome. Here, we describe a rapid, efficient, PCR-based recombineering method, FRUIT, that can be used to introduce scar-free point mutations, deletions, epitope tags, and promoters into the genomes of enteric bacteria. The efficiency of FRUIT is far higher than that of the most widely-used recombineering method for *Escherichia coli*. We have used FRUIT to introduce point mutations and epitope tags into the chromosomes of *E. coli* K-12, Enterotoxigenic *E. coli*, and *Salmonella enterica*. We have also used FRUIT to introduce constitutive and inducible promoters into the chromosome of *E. coli* K-12. Thus, FRUIT is a versatile, efficient recombineering approach that can be applied in multiple species of enteric bacteria.

## Introduction

Chromosomal mutagenesis is a critical genetic tool for the study of bacterial systems. Many bacteria cannot be readily transformed with linear DNA fragments, greatly limiting our ability to introduce chromosomal mutations. Recombineering, a method that involves expression of bacteriophage recombination proteins, has transformed our ability to engineer bacterial chromosomes using linear dsDNA (typically generated by PCR) or ssDNA (oligonucleotides) [1]. Thus, it is now possible to rapidly introduce point mutations, insertions, gene deletions, and epitope tags into the chromosomes of many bacterial species.

Existing recombineering methods involve two key components: (i) expression of bacteriophage recombination proteins, and (ii) generation of suitable DNA fragments for recombination. The latter component typically relies on specific DNA templates for PCR-based synthesis of dsDNA. Most described recombineering systems vary only in the DNA templates used, i.e. different selectable markers. Despite the wide variety of recombineering systems now available for enterobacteria such as *Escherichia coli* and *Salmonella enterica*, many have important limitations. First, some methods permit only imprecise excision of the selectable marker, leaving a 50–100 bp “scar” that can be problematic for future recombineering in the same strain and can alter expression of neighboring genes. Second, the efficiency of some methods is not sufficiently high to guarantee success with every attempted recombineering experiment. Third, some methods are limited to a single application, e.g. gene deletion. Here, we describe a highly efficient, rapid recombineering method, “Flexible Recombineering Using Integration of *thyA*” (FRUIT), that overcomes all of these limitations. FRUIT uses the *thyA* gene as both a selectable and counter-selectable marker, allowing for scar-free mutagenesis using a similar framework to previously-described recombineering methods. We have further developed FRUIT to allow for straightforward integration of any DNA sequence by combining recombineering with homologous recombination. Using these approaches, we have successfully introduced point mutations, gene deletions, epitope tags and artificial promoters into the genomes of *E. coli* K-12, Enterotoxigenic *E. coli* (ETEC), and *S. enterica* serovar Typhimurium.

## Materials and Methods

### Strains and Plasmids

All strains and plasmids used in this work are listed in Table 1. All oligonucleotides used for standard strain construction (i.e. not FRUIT) and plasmid cloning are listed in Table S1. *E. coli* K-12 MG1655 Δ*thyA* (AMD052) was constructed by electroporating an oligonucleotide, JW463, which has sequence immediately upstream and downstream of *thyA*, into MG1655 [2] containing pKD46 and grown in LB containing ampicillin and 0.2% arabinose to induce expression of the λ Red genes. Cells were recovered at 37°C for one hour and plated at 37°C onto M9 minimal medium containing 100 µg/ml thymine and 20 µg/ml trimethoprim. Recombinants were restreaked and then confirmed using colony PCR with primers flanking the expected site of *thyA* deletion. Colony PCR products were then sequenced. ETEC strain H10407 Δ*thyA* (AY001) was constructed similarly but using a PCR product containing sequence flanking *thyA*, amplified from MG1655 Δ*thyA* (AMD052) with oligonucleotides JW472+ JW473, and strain H10407 containing pKD46. *S. enterica* serovar Typhimurium strain 14028s Δ*thyA* was constructed similarly but using a PCR product with sequence flanking *thyA* generated by SOEing PCR [3] with oligonucleotides JW1189, JW1190, JW1191+ JW1192. All strain construction using FRUIT was as described below.

**Table 1:** List of strains and plasmids.

*Escherichia coli* strains			
MG1655		F-, λ^−^, *ilvG^−^*, *rfb-*50, *rph*-1	[2]
AMD052		MG1655 Δ*thyA*	This work
AMD095		MG1655 *lacZ* 2017-2019 GAT→TGA	This work
AMD225		MG1655 *allR*-FLAG_3_	This work
VS003		MG1655 Δ*thyA* P_high_:*lacZ*	This work
VS004		MG1655 Δ*thyA* P_med_:*lacZ*	This work
VS005		MG1655 Δ*thyA* P_low_:*lacZ*	This work
VS006		MG1655 Δ*thyA* P_rha_:*lacZ*	This work
ETEC strains			
H10407		Wild-type	[13]
AY001		H10407 Δ*thyA*	This work
AY004		H10407 *eslA* LexA site mutation	This work
AMD248		H10407 *allR*-FLAG_3_	This work
*Salmonella enterica* serovar Typhimurium strains			
14028s	Wild-type		[14]
AMD212	14028s Δ*thyA*		This work
JJA001	14028s Δ*oafA*		This work
BLP006	14028s *hilD*-FLAG_3_		This work
Plasmids			
pKD46	Encodes λ recombinase system		[10]
pKD13	*kan* ^r^ recombineering template		[10]
pGEM-T	T-tailed cloning vector		Promega
pAMD001	pGEM-T-*thyA*		This work
pAMD135	pGEM-T-FLAG_3_-*thyA*-FLAG_3_		This work
pVS003	pGEM-T-P_high_-*thyA*-P_high_		This work
pVS004	pGEM-T-P_med_-*thyA*-P_med_		This work
PVS005	pGEM-T-P_low_-*thyA*-P_low_		This work
pVS006	pGEM-T-P_rha_-*thyA*-P_rha_		This work

pAMD001 was constructed by PCR amplifying *thyA* from *E. coli* K-12 MG1655 using oligonucleotides JW495+ JW496, and ligating into pre-cut pGEM-T plasmid (Promega). Oligonucleotide JW495 includes a constitutive promoter [4]. For construction of pAMD134, duplicate sets of 3×FLAG tags were colony PCR amplified from an SPA-tagged strain of *E. coli* [5] with oligonucleotides JW1137+ JW1138, and JW1139+ JW1140, and cloned as *Apa*I-*Nco*I and *Sal*I-*Sac*I fragments upstream and downstream of *thyA* in pAMD001. pVS006 was constructed similarly except that the oligonucleotides used were JW2476+ JW2352 and JW2353+ JW2478 (for the pieces cloned upstream and downstream of *thyA*), the template was a colony of MG1655 *E. coli* K-12, and the restriction sites used were *Nco*I-*Sac*II and *Spe*I-*Sal*I. For construction of pVS003, the strong, constitutive promoter in pAMD001 was amplified by PCR using oligonucleotides JW2344+ JW2475, and cloned into pAMD001, downstream of *thyA*, as a *Spe*I-*Sal*I fragment. For construction of pVS004, *thyA* and its promoter were amplified in a single fragment from pAMD001 but incorporating a single base change in the extended −10 hexamer, using oligonucleotides JW2348+ JW2350. This PCR product was ligated into pre-cut pGEM-T. This plasmid served as a template for a PCR with oligonucleotides JW2344+ JW2475, which amplified the medium strength, constitutive promoter. This was cloned as a *Spe*I-*Sal*I fragment downstream of *thyA* in the same plasmid. pVS005 was cloned similarly except that oligonucleotide JW2348 was replaced with JW2349.

### FRUIT for Introducing Chromosomal Point Mutations, Custom Insertions, or Deletions

All oligonucleotides used for FRUIT are listed in Table S2. The *thyA* cassette (includes a strong, constitutive promoter) was amplified using primers with ∼40 nt 5′ sequence that matched the desired site of recombination. We refer to these primers as “Targeting upstream” and “Targeting downstream”. PCR products were purified using a minElute PCR purification kit (Qiagen) and electroporated into Δ*thyA* cells (AMD052, AY001 or AMD212) containing pKD46 and grown in LB containing ampicillin, 100 µg/ml thymine, and 0.2% arabinose to induce expression of the λ Red genes. Cells were recovered at 37°C for one hour and plated at 30°C onto M9 minimal medium (lacking thymine) containing ampicillin. Recombinants were restreaked and then confirmed using colony PCR with primers flanking the expected site of *thyA* insertion. We refer to these strains as “*thyA* ^+^ intermediate”. The desired chromosomal sequence was synthesized by SOEing PCR [3] using primers we refer to as “Flanking upstream”, “Flanking downstream”, “Mutagenesis upstream” and “Mutagenesis downstream”. PCR products were purified using a minElute PCR purification kit (Qiagen), and electroporated into *thyA* ^+^ intermediate cells containing pKD46 and grown in LB containing ampicillin and 0.2% arabinose to induce expression of the λ Red genes. Cells were recovered at 37°C for one hour and plated at 30°C onto M9 minimal medium containing 100 µg/ml thymine, 20 µg/ml trimethoprim and ampicillin. Recombinants were restreaked and then confirmed using colony PCR with primers flanking the expect site of mutagenesis. Colony PCR products were then sequenced. In some cases, *thyA* was reintroduced at its native locus. Specifically, the *thyA* gene and surrounding sequence was amplified from MG1655 (*thyA* ^+^) by colony PCR with oligonucleotides JW472+ JW473. PCR products were purified using a minElute PCR purification kit (Qiagen) and electroporated into Δ*thyA* recombinants containing pKD46 and grown in LB containing ampicillin, 100 µg/ml thymine, and 0.2% arabinose to induce expression of the λ Red genes. Cells were recovered at 37°C for one hour and plated at 37°C onto M9 minimal medium (lacking thymine and antibiotic). Recombinants were restreaked and restoration of the native *thyA* locus was confirmed using colony PCR with primers flanking the expected site of *thyA* insertion. For strains in which *thyA* was not reintroduced, pKD46 was lost by passaging at 37°C without antibiotic selection. Δ*thyA* strains were routinely grown in LB containing 100 µg/ml thymine since LB lacking thymine does not typically support growth of these strains.

### FRUIT for Introducing FLAG Tags or Promoter Sequences

*thyA* ^+^ intermediate strains were constructed and validated as described above except that the *thyA* cassette was amplified from the relevant tag or promoter plasmid. *thyA* ^+^ intermediate cells were grown to an OD_600_ of ∼1.0 in LB containing ampicillin. 100 µL cells were plated at 30°C onto M9 minimal medium containing 100 µg/ml thymine, 20 µg/ml trimethoprim and ampicillin. Recombinants were restreaked and then confirmed using colony PCR with primers flanking the expect site of mutagenesis. Colony PCR products were then sequenced. When desired, *thyA* was reintroduced at its native locus as described above.

### β-galactosidase Assay

2–3 ml cells were grown in LB at 37°C to an OD_600_ of 0.7–0.9 and the OD_600_ was recorded. Where indicated, 1 mM Isopropyl β-D-1-thiogalactopyranoside (IPTG) was added to cells during growth. 800 µL cells were pelleted at full speed in a microcentrifuge for 1 minute. Cell pellets were resuspended in 800 µL Z buffer (0.06 M Na_2_HPO_4_, 0.04 M NaH_2_PO_4_, 0.01 M KCl, 0.001 M MgSO_4_) +50 mM β-mercaptoethanol (added fresh). 20 µL chloroform and 10 µL 0.1% SDS were added to the cells followed by vortexing for 5 seconds. Assays were started by addition of 160 µL ONPG (4 mg/ml in dH_2_O) and stopped by addition of 400 µL 1 M Na_2_CO_3_, upon development of an appropriate yellow color. The reaction time was noted. Samples were centrifuged at full speed in a microcentrifuge to pellet the chloroform and any remaining cell debris. The OD_420_ of the supernatant was recorded. Assay units were calculated as A420/(A600)(total time).

### Chromatin Immunoprecipitation (ChIP)/qPCR

The ChIP method was based on an earlier study [6]. 40 ml *E. coli* K-12 (MG1655 or AMD225) or ETEC cells (H10407 or AMD248) were grown in LB at 37°C to an OD_600_ of 0.6–0.8. For *Salmonella enterica* serovar Typhimurium, 40 ml cells (BLP006 or 14028s) were grown in LB at 37°C to an OD_600_ of ∼1.0. Cells were crosslinked for 20 minutes with formaldehyde (1% final concentration), pelleted by centrifugation and washed once with Tris-buffered saline (TBS). Cell pellets were resuspended in 1 ml FA lysis buffer (50 mM Hepes-KOH, pH 7, 150 mM NaCl, 1 mM EDTA, 1% Triton X-100, 0.1% sodium deoxycholate, 0.1% SDS) with 2 mg/ml lysozyme and incubated at 37°C for 30 minutes. Samples were then chilled and sonicated for 30 minutes in a Bioruptor sonicator (Diagenode) with 30 s on/30 s off pulsing at maximum amplitude. Samples were pelleted in a microcentrifuge to remove debris and supernatants (“chromatin”) were saved. 1 ml FA lysis buffer was added to chromatin samples, and these were stored indefinitely at −20°C. For each immunoprecipitation (IP), 500 µL chromatin was incubated with 300 µL FA lysis buffer, 20 µL Protein A Sepharose slurry (50%) in TBS and either 5 µL anti-LexA antibody (Santa Cruz Biotechnology) or 2 µL M2 anti-FLAG antibody (Sigma) for 90 minutes at room temperature with gentle mixing on a rotisserie rotator. Beads were then pelleted at 1,500×g in a microcentrifuge for 1 minute. The supernatant was removed and the beads were resuspended in 750 µL FA lysis buffer and transferred to a Spin-X column (Corning). Beads were then incubated for 3 minutes with gentle mixing on a rotisserie rotator before being pelleted at 1,500×g in a microcentrifuge for 1 minute. Equivalent washes were performed with FA lysis buffer, high salt FA lysis buffer (50 mM Hepes-KOH, pH 7, 500 mM NaCl, 1 mM EDTA, 1% Triton X-100, 0.1% sodium deoxycholate, 0.1% SDS), ChIP wash buffer (10 mM Tris-HCl, pH 8.0, 250 mM LiCl, 1 mM EDTA, 0.5% Nonidet-P40, 0.5% sodium deoxycholate) and TE (10 mM Tris-HCl, pH 7.5, 1 mM EDTA). After the TE wash, beads were transferred to a fresh Spin-X column and eluted with 100 µL ChIP elution buffer (50 mM Tris-HCl, pH 7.5, 10 mM EDTA, 1% SDS) for 10 minutes at 65°C with occasional agitation. Eluted samples were centrifuged at 1,500×g in a microcentrifuge for 1 minute. Supernatants were decrosslinked by boiling for 10 minutes and purified using a PCR purification kit (Qiagen). For all ChIP/qPCR experiments, 20 µL chromatin was decrosslinked by boiling for 10 minutes and purified using a PCR purification kit (Qiagen). This sample served as the “input” control.

For qPCR, ChIP and input samples were analyzed using an ABI 7500 Fast real time PCR machine, as described previously [7]. Enrichment of ChIP samples was calculated relative to a control region within the transcriptionally silent *bglG* gene for *E. coli* K-12/ETEC, and within the *sbcC* gene for *S.* Typhimurium. Values were normalized to those for input DNA. Occupancy units represent background-subtracted fold-enrichment relative to the control region. Oligonucleotides used for real time PCR with *E. coli* K-12 samples were JW125+ JW126 (*bglB*), JW1296+ JW1297 (*allA*), JW0090+ JW0091 (*galE*), and JW0416+ JW0417 (*purR*). Oligonucleotides used for real time PCR with ETEC samples were JW125+ JW126 (*bglB*), JW2197+ JW2198 (*sulA*), and JW741+ JW742 (*eslA*). Oligonucleotides used for real time PCR with *S*. Typhimurium samples were JW1495+ JW1496 (*sbcC*), JW2432+ JW2433 (*prgH*), and JW2444+ JW2445 (*invH*).

### Soft Agar Motility Assay

Soft agar motility assays were performed as described previously [8].

### Western Blot

For each sample analyzed, 20 µL sonicated, crosslinked cell extract from a ChIP experiment was separated on a 4–20% acrylamide gradient gel (Bio-Rad). Proteins were transferred to PVDF membrane and probed with M2 anti-FLAG antibody (Sigma; 1 in 2,000 dilution) and HRP-conjugated goat anti-mouse antibody (1 in 100,000 dilution). Tagged proteins were visualized using the ImmunStar WesternC kit (Bio-Rad).

### Comparison of FRUIT and pKD13 Recombineering

*thyA* was used to replace the *yacL* gene in MG1655 (*E. coli* K-12) using FRUIT. The site of *thyA* insertion is identical to that of the site of insertion of the *kan* ^R^ cassette from pKD13 that was used to construct the Δ*yacL* strain in the Keio deletion collection [9]. Recombineering templates for *thyA* and *kan* ^R^ were then generated by PCR amplification from the Δ*yacL* strains in which *yacL* was replaced with *thyA* or *kan* ^R^, respectively. PCR products were checked by agarose gel electrophoresis and quantified. Equimolar amounts of PCR product for the *thyA* and *kan* ^R^ PCRs were mixed and used for recombineering into MG1655 Δ*thyA* expressing λ Red recombineering proteins from the pKD46 plasmid [10]. Colonies were verified for introduction of the *thyA* or *kan* ^R^ cassette at the correct location using colony PCR with primers flanking the expected site of insertion (oligonucleotides JW3017+ JW3018). At least 8 colonies were tested for every recombineering experiment.

## Results

### Overview of FRUIT

**Figure 1:**
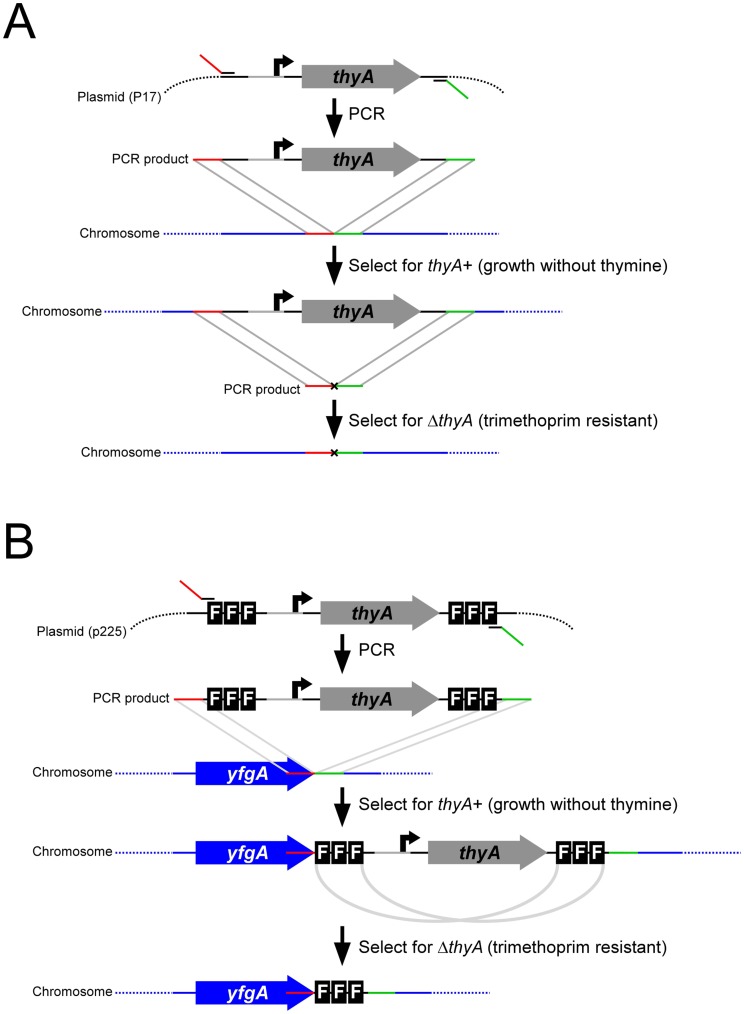
Schematic of FRUIT method. (A) Schematic of FRUIT for introducing point mutations or deletions. PCR product is amplified from the recombineering template plasmid (pAMD001), incorporating flanking sequence with identity to the desired site of recombination. This PCR product is introduced into cells expressing λ recombinase proteins and recombinants are selected using the *thyA* marker (growth on media lacking thymine). A mutation can then be introduced by recombineering a second PCR product, selecting for recombinants using counter-selection of *thyA* (growth in the presence of trimethoprim). (B) Schematic of FRUIT for introducing FLAG tags. As above, except that loss of *thyA* occurs spontaneously due to homologous recombination of duplicate sets of FLAG tags.

FRUIT uses *thyA* as a selectable and counter-selectable marker, as described previously for BAC mutagenesis in *E. coli* [11]. *thyA* is a widely-conserved bacterial gene, required for the production of thymine, an essential nutrient. In cells otherwise lacking a copy of the *thyA* gene, chromosomal recombination of DNA fragments containing *thyA* can be selected for by growth on minimal media lacking thymine. Counter-selection of *thyA* requires growth on media containing trimethoprim. Trimethoprim is an inhibitor of dihydrofolate reductase, an enzyme that recycles tetrahydrofolate. Tetrahydrofolate is an essential cofactor that is depleted by ThyA. Hence, ThyA is toxic in cells treated with trimethoprim, due to depletion of tetrahydrofolate. The basic FRUIT method (Figure 1) involves recombination of a *thyA*-containing PCR product into the chromosome of Δ*thyA* cells expressing λ phage Red recombination proteins. Successful recombinants are selected on medium lacking thymine. Clean replacement of the *thyA* marker is achieved either by λ Red recombination of a PCR product that lacks a marker (Figure 1A), or homologous recombination of sequences introduced by the original recombineering step (Figure 1B). In theory, the FRUIT method can be applied to any bacterium with (i) a functional *thyA* gene, and (ii) a described system for expression of bacteriophage recombination proteins. Thus, we have used FRUIT to introduce point mutations, gene deletions (Figure 1A), epitope tags and artificial promoters (Figure 1B) into the chromosomes of *E. coli* K-12, Enterotoxigenic *E. coli*, and *S. enterica* serovar Typhimurium.

### Introducing Point Mutations and Gene Deletions Using FRUIT

We tested FRUIT in the MG1655 strain of *E. coli* K-12. We first precisely deleted the chromosomal copy of *thyA* using oligonucleotide recombineering [12] with counter-selection on medium containing trimethoprim. We confirmed the deletion of *thyA* by sequencing of a PCR product across the junction generated by deletion. We then cloned *thyA* onto a plasmid under the control of an artificial, constitutively-transcribed promoter [4]. This plasmid served as a template for PCR amplification of *thyA* and its promoter using primers that included ∼40 nt sequence identity to the desired site of chromosomal recombination. All FRUIT experiments described hereafter begin with amplification of such a PCR product, electroporation of the PCR product into Δ*thyA* cells expressing λ Red proteins from pKD46 [10], and selection of recombinants on minimal medium lacking thymine. Recombinants were validated by colony PCR; the success rate was close to 100% in all cases. For introduction of point mutations and gene deletions, a second recombineering step was utilized. This involved generating a second PCR product with appropriate sequence identity to the planned site of recombination but containing no selectable marker. This PCR product was electroporated into recombinants from the first FRUIT step (still expressing λ Red recombination proteins) and recombinants lacking *thyA* were selected on medium containing trimethoprim. Recombinants were validated by colony PCR; approximately 30–50% of colonies are successful recombinants in a typical FRUIT experiment, with the rest presumably gaining resistance to trimethoprim through other mutations such as point mutations, insertions or deletions in *thyA* that disrupt ThyA function.

**Figure 2:**
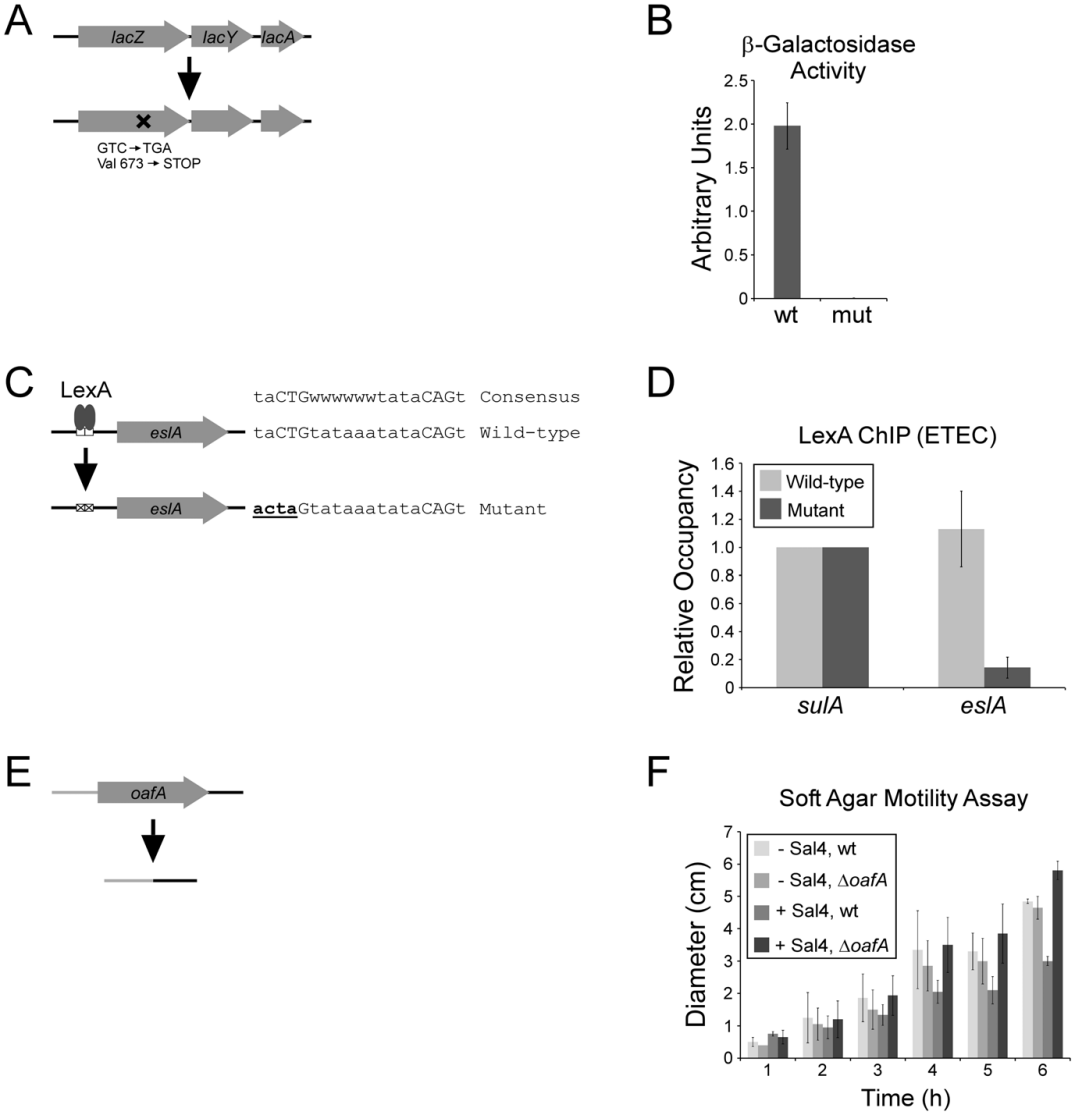
FRUIT mutagenesis of MG1655 (*E. coli* K-12) *lacZ*, H10407 (ETEC) *eslA*, and 14028s (*S. enterica* serovar Typhimurium) *oafA*. (A) Schematic indicating the mutation within *lacZ*. (B) β-galactosidase assay in wild-type MG1655 and mutant MG1655 with a stop codon introduced within *lacZ*. (C) Schematic inidicating the mutation in the putative LexA site. (D) ChIP/qPCR assay to measure association of LexA with the region upstream of *sulA* (known LexA site) and the region upstream of *eslA* in wild-type and mutant strains. Relative occupancy values represent background-subtracted enrichment relative to that upstream of *sulA*. (E) Schematic indicating the deletion of *oafA*. (F) Soft agar motility assay of wild-type or mutant strains in the presence or absence of Sal4 antibody. Values indicate the diameter of the halo of motile cells after the indicated time.

As a first application of FRUIT, we introduced a three base pair point mutation into the *E. coli* K-12 *lacZ* gene, resulting in premature translation termination (Figure 2A). Using β-galactosidase assays of wild-type and mutant cells induced with IPTG we demonstrated that the mutation resulted in a drastic reduction in functional LacZ protein (Figure 2B).

Having demonstrated FRUIT in a laboratory strain, we wished to test its utility in a clinical isolate. For this purpose we selected the H10407 strain of ETEC. H10407 is closely related to *E. coli* K-12 and their genomes are largely co-linear [13]. We first precisely deleted the chromosomal copy of *thyA* using recombineering of a PCR product generated using a colony of *E. coli* K-12 Δ*thyA* as a template. Recombinants were isolated by counter-selection of *thyA* on medium containing trimethoprim. We confirmed the deletion of *thyA* by sequencing of a PCR product across the junction generated by deletion. In a separate study, we identified a putative binding site for the transcription factor LexA upstream of a predicted gene that has no close homologue in *E. coli* K-12 (Figure 2C). We named this gene *eslA* (ETEC-specific LexA-regulated gene A). We demonstrated robust association of LexA with this putative site using ChIP and quantitative real time PCR (ChIP/qPCR; Figure 2D). We used FRUIT to introduce a four base pair mutation into the putative LexA site, disrupting two of the three bases in the CTG motif that is critical for association of LexA (Figure 2C). Using ChIP/qPCR we demonstrated that this mutation results in a dramatic decrease in association of LexA relative to that at a site upstream of *sulA* (Figure 2D).

We next wished to test FRUIT in an enteric pathogen that is more distantly related to *E. coli* K-12 than ETEC. For this, we selected the 14028s strain of *S. enterica* serovar Typhimurium, a clinical isolate [14]. We first precisely deleted the chromosomal copy of *thyA* using recombineering of a PCR product generated by SOEing [3]. Recombinants were isolated by counter-selection of *thyA* on medium containing trimethoprim. We confirmed deletion of *thyA* by sequencing of a PCR product across the junction generated by deletion. In a separate study, we wished to delete the *oafA* gene that encodes encodes an enzyme that modifies the O-antigen. Using FRUIT, we constructed a clean deletion of the *oafA* gene (Figure 2E). Treatment of wild-type *S.* Typhimurium with the Sal4 antibody results in motility arrest due to binding of Sal4 to the O-antigen [8]. *oafA* is required for Sal4 to bind *S.* Typhimurium cells and arrest motility [8]. We tested the motility of the wild-type and Δ*oafA* strains +/− Sal4, using a soft agar motility assay. As expected, motility of wild-type but not Δ*oafA* cells was significantly reduced by the addition of Sal4 (Figure 2F).

### Introducing Epitope Tags Using FRUIT

**Figure 3:**
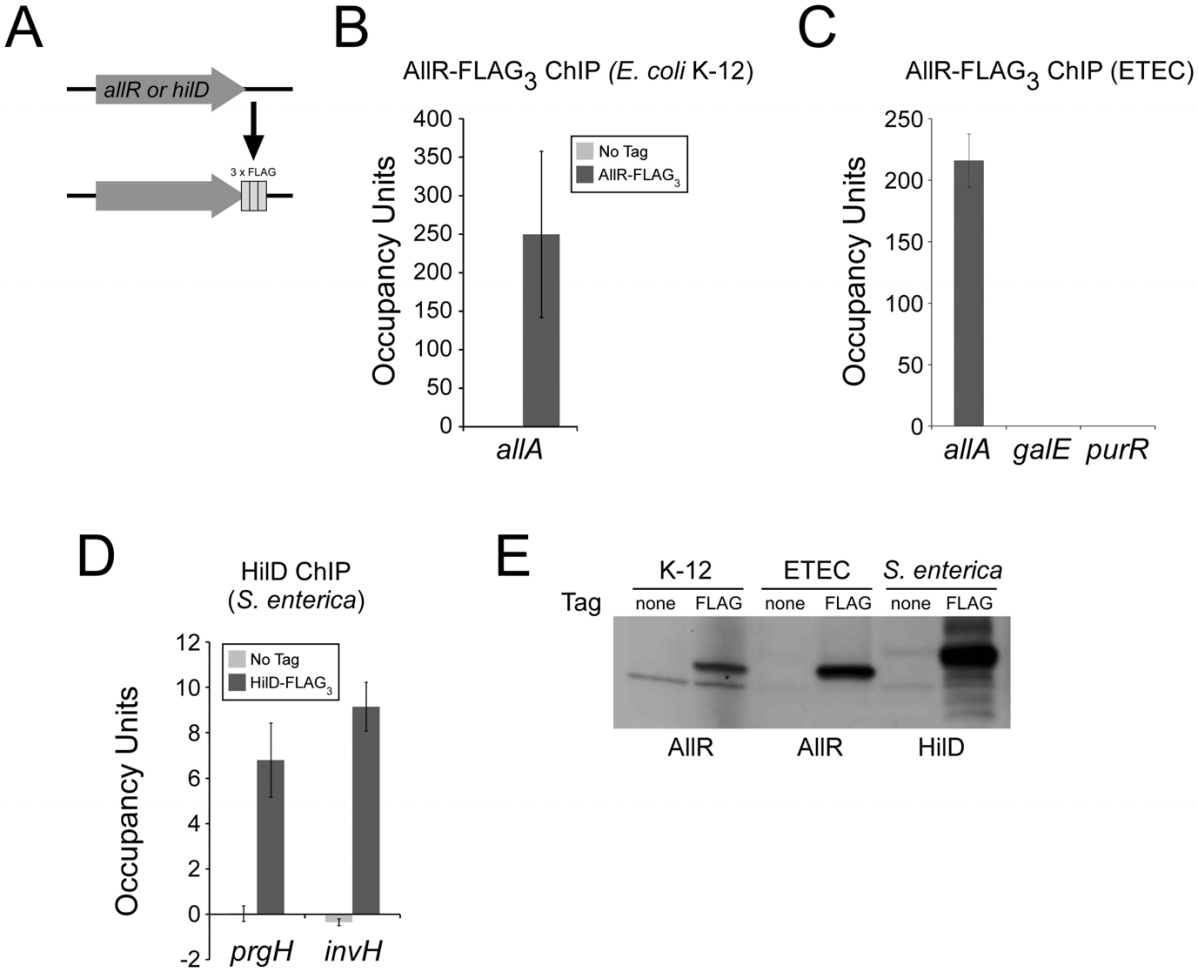
FRUIT epitope-tagging of MG1655 (*E. coli* K-12) *allR*, H10407 (ETEC) *allR*, and 14028s (*S. enterica* serovar Typhimurium) HilD. (A) Schematic indicating C-terminal tagging with three FLAG tags. (B) ChIP/qPCR assay to measure association of MG1655 AllR-FLAG_3_ with the region upstream of *allA* (known AllR site in *E. coli* K-12) [32]. Values are also shown for a control ChIP with an untagged strain. Occupancy unit values represent background-subtracted enrichment relative to a control region. (C) ChIP/qPCR assay to measure association of H10407 AllR-FLAG_3_ with the region upstream of *allA*, or with predicted non-target regions upstream of *galE* and *purR*. Occupancy unit values represent background-subtracted enrichment relative to a control region. (D) ChIP/qPCR assay to measure association of 14028s HilD-FLAG_3_ with the regions upstream of *prgH* and *invH* (known HilD targets) [33]. Values are also shown for a control ChIP with an untagged strain. Occupancy unit values represent background-subtracted enrichment relative to a control region. (E) Western blot probing extracts from untagged and FLAG-tagged strains for MG1655 (K-12), H10407 (ETEC) and 14028s (*S. enterica*). Note that the anti-FLAG antibody cross-reacts with a protein expressed *E. coli* K-12.

Introduction of point mutations and gene deletions using FRUIT requires two recombineering steps: one to introduce the *thyA* marker and one to remove it. This process is analogous to the *delitto perfetto* method of chromosomal mutagenesis in the yeast, *Saccharomyces cerevisiae*, that employs *URA3* as a selectable and counter-selectable marker [15]. There are also many systems in yeast for epitope tagging in which the *URA3* marker is introduced and then spontaneously resolved due to homologous recombination of duplicate sets of epitope tags on either side of the marker [16]. Inspired by this approach, we created a plasmid that contains *thyA* under the control of an artificial promoter [4], flanked by two identical copies of three FLAG tags (Figure 1B). We reasoned that, following recombineering of the FLAG_3_-*thyA*-FLAG_3_ cassette into a bacterial chromosome, homologous recombination of the two sets of FLAG tags would occur spontaneously at a low frequency and could be selected for by growth on medium containing trimethoprim, due to loss of *thyA* (Figure 1B). We used this method to introduce FLAG tags at the C-terminus of the transcription factors AllR in *E. coli* K-12 and ETEC, and HilD in *S.* Typhimurium (Figure 3A). In each case, the frequency of successful recombination of the two sets of tags was sufficiently high to isolate tens of recombinants. These were checked by sequencing of a PCR product surrounding the FLAG tags. We then used ChIP/qPCR to measure association of the transcription factors with known target sites in their respective genomes (Figure 3B–D). In each case, we detected robust enrichment of the known target site. For ETEC AllR we tested association with two non-target sites (*galE* and *purR*) and detected no significant enrichment (Figure 3C). For *E. coli* K-12 AllR and *S.* Typhimurium HilD we did not detect enrichment of known target sites when using an untagged strain (Figure 3B+D). Lastly, we were able to detect the tagged proteins from all three species by Western blot (Figure 3E).

### Promoter Replacement Using FRUIT

**Figure 4:**
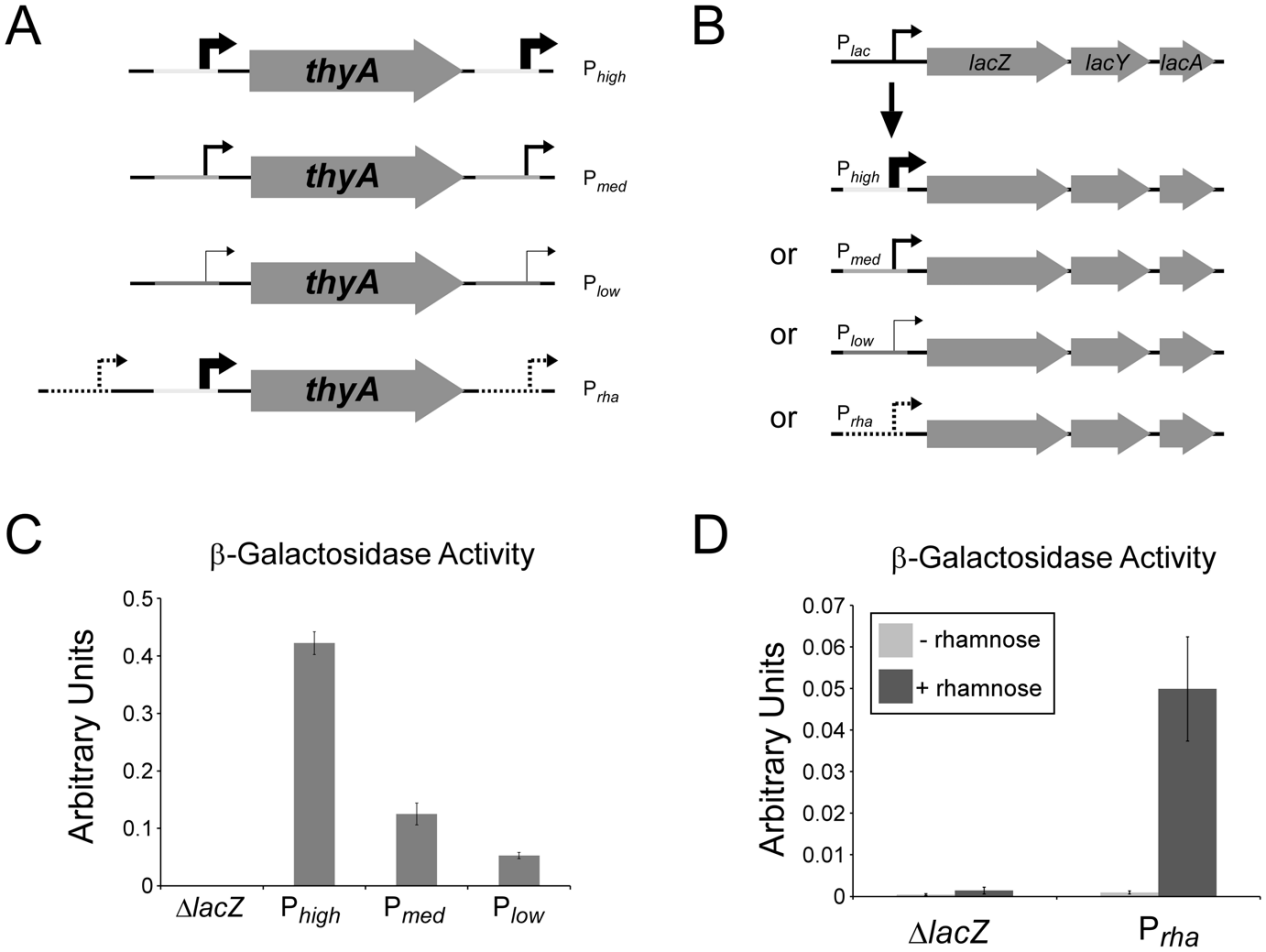
FRUIT promoter swaps in MG1655 (*E. coli* K-12). (A) Schematic indicating the plasmid templates used for FRUIT. (B) Schematic indicating replacement of the *lacZYA* promoter with P_high_, P_med_, P_low_ or P_rha_ promoters. (C) β-galactosidase assay in Δ*lacZ* MG1655 and mutant strains with P_high_, P_med_ or P_low_ driving expression of *lacZYA* (cells were grown without IPTG). (D) β-galactosidase assay in Δ*lacZ* MG1655 and a mutant strain with P_rha_ driving expression of *lacZYA*. Assays were performed ± rhamnose.

We reasoned that any sequence could be introduced into chromosomal DNA using FRUIT using a method equivalent to that described above for epitope tags. We constructed a plasmid containing *thyA* flanked by identical copies of a strong, constitutive promoter. The upstream copy of the promoter was positioned such that it drives transcription of *thyA*. We then constructed two derivatives of this plasmid in which the extended −10 sequence in both copies of the constitutive promoter was mutated from TG to either CG or CT (Figure 4A). This promoter is expected to have high, medium or low strength with a TG, CG, or CT respectively at this position [4]. We also constructed a plasmid that contains *thyA* and its promoter flanked by identical copies of the *rhaBAD* promoter whose transcription is induced by the sugar, rhamnose (Figure 4A) [17]. We used FRUIT to introduce all constructs upstream of the *lacZYA* operon in *E. coli* K-12, simultaneously replacing the natural promoter (Figure 4B). The efficiency was similar to that observed for introducing epitope tags. Strains were checked by sequencing of a PCR product surrounding the new promoters. We then confirmed the effect of these promoters by performing β-galactosidase assays which measure the level of LacZ. As expected, the high, medium and low strength promoters resulted in high, medium and low levels of β-galactosidase activity, respectively (Figure 4C). Furthermore, the *rhaBAD* promoter resulted in rhamnose-dependent β-galactosidase activity (Figure 4D).

### Efficiency of FRUIT Compared to Recombineering with Kanamycin Resistance Selection

The most widely used recombineering method involves PCR amplification of a kanamycin resistance or chloramphenicol resistance gene with ∼40 nt flanking sequence to direct recombination to the desired location [10]. The antibiotic resistance gene can then be removed by expressing *flp* recombinase, leaving a ∼80 nt scar. We have previously used this approach to delete genes in *E. coli* K-12 using plasmid pKD13 (contains a kanamycin resistance gene) as a recombineering template [18]. Furthermore, this method was used to construct a near-complete gene deletion collection for *E. coli* K-12 [9]. Nonetheless, we have found this method to be inefficient, often generating no successful recombinants. We directly compared the efficiency of gene replacement with FRUIT to that with pKD13. We generated PCR products containing the kanamycin resistance gene (from pKD13) or *thyA*. These PCR products had 43 bp (short), 134 bp (medium), or 210 bp (long) flanking sequence on each side of the selectable marker; the flanking sequence was identical to sequence flanking the *E. coli* K-12 *yacL* gene. For each of the short, medium and long PCRs, we mixed equimolar amounts of the pKD13 and *thyA* products and electroporated the mixture into *E. coli* K-12 expressing the λ Red recombinase proteins. After recovering the cells we plated half onto medium containing kanamycin and half onto minimal medium lacking thymine. Eight colonies were selected from each plate and validated using colony PCR with primers flanking the site of insertion. Representative efficiencies of each method are listed in Table 2. Regardless of the length of flanking sequence, FRUIT was at least 30-fold more efficient than pKD13. Furthermore, 100% of recombinants generated by FRUIT were validated by colony PCR whereas recombinants generated using pKD13 were often incorrect, presumably due to recombination of the kanamycin resistance gene with an alternative locus.

**Table 2:** Comparison of FRUIT to pKD13-mediated recombineering.

	FRUIT			pKD13			
Length ofArms (bp)	Number ofColonies	PCR CheckFrequency^a^	RecombineeringFrequency^b^	Number ofColonies	PCR CheckFrequency^a^	RecombineeringFrequency^b^	Ratio(FRUIT/pKD13)^c^
43	1580	1	1.05E−04	26	0.125	2.17E−07	486.15
134	3350	1	2.23E−04	117	0.75	5.85E−06	38.18
210	1990	1	1.33E−04	14	1	9.33E−07	142.14

a Frequency with which candidate colonies were successfully verified.

b Number of colonies/number of viable cells.

c Relative efficiency of FRUIT as compared to pKD13.

## Discussion

There are many described methods for recombineering in *E. coli* and *S. enterica*. The most commonly used method for gene deletion is that described by Datsenko and Wanner [10]. Although this method has been used successfully in a wide range of enterobacterial species, it cannot be used to make point mutations, introduce epitope tags or promoters, and it leaves a ∼80 bp scar. Importantly, FRUIT uses the same plasmid (pKD46) to express λ recombinase proteins as that used by Datsenko and Wanner; the key improvement in the use of *thyA* rather than *kan* ^R^ in the recombineering templates. The use of *thyA* allows for selection and subsequent counter-selection; hence, FRUIT can be used to make scarless mutations of any type. FRUIT is also >30-times more efficient than the method described by Datsenko and Wanner (Table 2). We have not been able to determine why FRUIT is so much more efficient. Given that the only difference between the two techniques is the marker used for selection, we propose that the choice of marker may have large effects on the efficiency of recombineering.

Recombineering using *thyA* has been described previously for BAC mutagenesis [11]. Hence, our work is an extension of prior studies using this marker. Similarly, other methods have been described that use recombineering substrates with marker genes or cassettes that can be both selected and counter-selected. These include use of *tolC* [19] and *galK* [20] as single-gene markers, and *tetAR* as a two-gene cassette [21]. Cassettes with separate selectable and counter-selectable markers have also been developed, e.g. chloramphenicol resistance gene and *sacB*, which can be counter-selected by growth on media containing sucrose [22]. Of particular note, several groups have used restriction of chromosomal DNA by I-SceI meganuclease as a counter-selection [23], [24], [25], [26], [27]. I-SceI cuts at a large recognition site that is not typically found in chromosomal DNA. Introducing a restriction site for I-SceI adjacent to a selectable marker creates a cassette that can be counter-selected by expression of I-SceI. Any recombineering method with an efficient counter-selection step could, in principle, be used identically to FRUIT. However, FRUIT is the first such method that has been adapted to allow for introduction of epitope tags or promoters. Only two other methods have been described that are designed specifically for the introduction of epitope tags by recombineering and neither uses a counter-selectable marker [28], [29]. Hence, both methods leave a chromosomal scar. There are no methods currently described for introducing heterologous promoters.

FRUIT can be easily adapted to recombineer sequences in addition to FLAG tags or promoters, using an analogous approach (Figure 1B). Equivalent methods are widely used to introduce sequences into yeast chromosomes. These sequences include a wide variety of epitope tags [30], reporter genes [31], and affinity tags [31]. In principle, recombineering templates could be created to allow for integration of any sequence by FRUIT using the method illustrated in Figure 1B.

The flexibility of FRUIT also applies to the bacterial species in which it is applied. We have tested FRUIT in three enterobacterial species. Given the high degree of conservation of *thyA*, we expect that FRUIT can be applied to many other species. This is especially important for species with low recombineering efficiencies, for which the method described by Datsenko and Wanner is ineffective [23].

In conclusion, we have developed a method for recombineering that combines the strengths of many existing approaches. We anticipate that FRUIT will be a widely-used method for introducing point mutations, deletions, epitope tags, heterologous promoter, and other commonly-used sequences into the chromosomes of a wide range of enterobacterial species.

## Supporting Information

Table S1 List of oligonucleotides used for strain and plasmid construction, and for comparison of recombineering methods (excludes oligonucleotides used for FRUIT).
(DOC)

Table S2 List of oligonucleotides used for FRUIT.
(DOC)
