# Supplementary material for: FRUIT, a Scar-Free System for Targeted Chromosomal Mutagenesis, Epitope Tagging, and Promoter Replacement in Escherichia coli and Salmonella enterica
Source: PLoS One. 2012 Sep 27;7(9):e44841. doi: 10.1371/journal.pone.0044841 (PMC3459970; doi:10.1371/journal.pone.0044841)
Supplement: Table S1 — List of oligonucleotides used for strain and plasmid construction, and for comparison of recombineering methods (excludes oligonucleotides used for FRUIT). (DOC) [file pone.0044841.s001.doc]

| **Name** | **Sequence** |
| --- | --- |
| JW090 | AAAAAGCGCAATCATTCA |
| JW091 | GCGAAAAGTGTGACATGG |
| JW125 | AAGCGAAAATCGGCAATA |
| JW126 | CATGGCCTGCAACATATC |
| JW416 | GTTTTTCCGGCGTACC |
| JW417 | TTCAGCTCCTGCAAAATC |
| JW463 | AGCCCACAGCAACACGTTTCCTGAGGAACCTTACGAAACATCCTGCCAGAGCCGACGCCA |
| JW472 | CCGACGCGCAGTTTA |
| JW473 | CACGTTGTGTTTTCATGC |
| JW495 | TAGACAGCTGCATGCATCTTTGTTATGGTGTGTTCATATGATAACGGTAATGAGGAACCATGAAACAGT |
| JW496 | GTGTAGGCTGGAGCTGTCAGGAAACGTGTTGC |
| JW576 | GCATAGTTTTCGCTGTAGTTTTCATCGTTCGCCGCTTTTTGACACCAGACCAACTGGTAA |
| JW741 | CGCATATCCGGTTATTCTAT |
| JW742 | TCGAGATCCCGGAGTAAT |
| JW1137 | AATAATGGGCCCGGCGGTGGCGACTACAAAGACCATGACGGTG |
| JW1138 | AATAATCCATGGCTACTTGTCATCGTCATCCTTG |
| JW1139 | AATAATGTCGACTACAAAGACCATGACGGTG |
| JW1140 | AATAATGAGCTCACTACTTGTCATCGTCATCCTTG |
| JW1189 | CAGCAACACGTTTCCTGAGGAACCGTTATGGCAGGATCGTCAGCGC |
| JW1190 | GCGCTGACGATCCTGCCATAACGGTTCCTCAGGAAACGTGTTGCTG |
| JW1191 | ATTGGCTATGGCGCGTTT |
| JW1192 | GCGGGCGGAAGAAAAATA |
| JW1296 | CCCTGTTCAATTTGTGGA |
| JW1297 | GGTCAGCTTGGTTTCAAT |
| JW1495 | ACCCGTTCGATGTTGTTA |
| JW1496 | TCTGCCCGTAAATCTCAG |
| JW2197 | TTCCGAAAATAGGGTTGA |
| JW2198 | GAACGACGAAGAACGATG |
| JW2344 | AGGCATACTAGTTAGACAGCTGCATGCATCT |
| JW2348 | TAGACAGCTGCATGCATCTTCGTTATGGTGTGTTCATATGAT |
| JW2349 | TAGACAGCTGCATGCATCTTCTTTATGGTGTGTTCATATGAT |
| JW2350 | GTGTAGGCTGGAGCTGTTAG |
| JW2352 | GATCAACCGCGGTACGACCAGTCTAAAAAGCG |
| JW2353 | AGCGATACTAGTTTCAGCAAATTGTGAACATC |
| JW2375 | CGGGCATTGCCTACTTAATTTCC |
| JW2432 | CTGGGCTTGTTATCGTCTTC |
| JW2433 | GTGCGGTAATCTGCTGCTAT |
| JW2444 | CATTCGCCCCCTTATAACTA |
| JW2445 | CTCCAACTACAGAAGAATGAGG |
| JW2475 | TAGCTAGTCGACAAACTTGGGGATTACCGTTATCATATGAACACAC |
| JW2476 | GGCATCCCATGGTTCAGCAAATTGTGAACATC |
| JW2478 | TAGCTAGTCGACAAACTTGGGGTACGACCAGTCTAAAAAGCG |
| JW3017 | CCGGAAGAGTACCAGACC |
| JW3018 | TTTACCCGCGACATTAAC |

**Table S1. List of oligonucleotides used for strain and plasmid construction, and for comparison of recombineering methods (excludes oligonucleotides used for FRUIT).**
