# Supplementary material for: FRUIT, a Scar-Free System for Targeted Chromosomal Mutagenesis, Epitope Tagging, and Promoter Replacement in Escherichia coli and Salmonella enterica
Source: PLoS One. 2012 Sep 27;7(9):e44841. doi: 10.1371/journal.pone.0044841 (PMC3459970; doi:10.1371/journal.pone.0044841)
Supplement: Table S2 — List of oligonucleotides used for FRUIT. (DOC) [file pone.0044841.s002.doc]

| **Name** | **Purpose** | **Sequence** |
| --- | --- | --- |
| JW1467 | HilD-FLAG_3_ Targeting upstream | TATTTTAAAACTACGCCATCGACATTCATAAAAATGGCGAACCATGGCGGTGGCGACTAC |
| JW1468 | HilD-FLAG_3_ Targeting downstream | TAAAAATCTTTACTTAAGTGACAGATACAAAAAATGTTAGTTGGGAGCTCACTACTTGTC |
| NM331 | Δ*oafA* Targeting upstream | CTTAATTTCGTCTTGTGTGGCACCTTGGAATTATAGGTAAAAATAGACAGCTGCATGCAT |
| NM332 | Δ*oafA* Targeting downstream | TGTTGTAGTTTTATAAAATAAAAAGAGGGGCAAGCCCCTCTGTTGTGTAGGCTGGAGCTG |
| NM362 | Δ*oafA* Mutagenesis upstream | GGCACCTTGGAATTATAGGTAAAAAACAGAGGGGCTTGCCCCTCTTTTTA |
| NM363 | Δ*oafA* Mutagenesis downstream | TAAAAAGAGGGGCAAGCCCCTCTGTTTTTTACCTATAATTCCAAGGTGCC |
| NM333 | Δ*oafA* Flanking upstream | CAAACCAGCAATGAGTCGTC |
| NM334 | Δ*oafA* Flanking downstream | AACGAGCACATGAACAGCAG |
| JW567 | *lacZ* mutant Targeting upstream | GGATGGTAAGCCGCTGGCAAGCGGTGAAGTGCCTCTGGATGTCTAGACAGCTGCATGCAT |
| JW568 | *lacZ* mutant Targeting downstream | TGCGGTAGTTCAGGCAGTTCAATCAACTGTTTACCTTGTGGAGCGTGTAGGCTGGAGCTG |
| JW565 | *lacZ* mutant Mutagenesis upstream | GGTGAAGTGCCTCTGGATTGAGCTCCACAAGGTAAACAGTT |
| JW566 | *lacZ* mutant Mutagenesis downstream | AACTGTTTACCTTGTGGAGCTCAATCCAGAGGCACTTCACC |
| JW569 | *lacZ* mutant Flanking upstream | GAAGCAAAACACCAGCAG |
| JW570 | *lacZ* mutant Flanking downstream | CAGCTCGATGCAAAAATC |
| JW737 | *eslA* mutant Targeting upstream | AGGTGATTCGCAAAACGTGCTGGATAGCAAACAAATTATTTGATAGACAGCTGCATGCAT |
| JW738 | *eslA* mutant Targeting downstream | TGCATCTTATCTTCCCCATGCAATAACTGTATATTTATACAGTAGTGTAGGCTGGAGCTG |
| JW739 | *eslA* mutant Mutagenesis upstream | GGATAGCAAACAAATTATTTGAACTAGTATAAATATACAGTTATTGCATGG |
| JW740 | *eslA* mutant Mutagenesis downstream | CCATGCAATAACTGTATATTTATACTAGTTCAAATAATTTGTTTGCTATCC |
| JW741 | *eslA* mutant Flanking upstream | CGCATATCCGGTTATTCTAT |
| JW742 | *eslA* mutant Flanking downstream | TCGAGATCCCGGAGTAAT |
| JW1303 | AllR-FLAG_3_ Targeting upstream | GCGAAAGACATCAGTACGGCGCTTGGGCTAAAACCCCCTGTAGCCGGCGGTGGCGACTAC |
| JW1304 | AllR-FLAG_3_ Targeting downstream | TTTAAAAAAGTCCCGCTCCAGCGAGCGGGATGCGGCTCAGTTGGGAGCTCACTACTTGTC |
| JW2495 | P_high_/P_med_/P_low_ Targeting upstream | GCAATTAATGTGAGTTAGCTCACTCATTAGGCACCCCAGGCTTCGCGGGATTTAGACAGC |
| JW2496 | P_high_/P_med_/P_low_ Targeting downstream | AGTGAATCCGTAATCATGGTCATAGCTGTTTCCTGTGAAACTTGGGGATTACCGTTATCA |
| JW2497 | P_rha_ Targeting upstream | CAATTAATGTGAGTTAGCTCACTCATTAGGCACCCCAGGCTTTCCCATGGTTCAGCAAAT |
| JW2498 | P_rha_ Targeting downstream | CCAGTGAATCCGTAATCATGGTCATAGCTGTTTCCTGTGAAACTTGGGGTACGACCAGTC |

**Table S2. List of oligonucleotides used for FRUIT.**
